# Supplementary material for: CRPC Membrane-Camouflaged, Biomimetic Nanosystem for Overcoming Castration-Resistant Prostate Cancer by Cellular Vehicle-Aided Tumor Targeting
Source: Int J Mol Sci. 2022 Mar 26;23(7):3623. doi: 10.3390/ijms23073623 (PMC8998744; doi:10.3390/ijms23073623)
Supplement: Supplementary file 1 [file ijms-23-03623-s001.zip › ijms-1636116-supplementary.pdf]

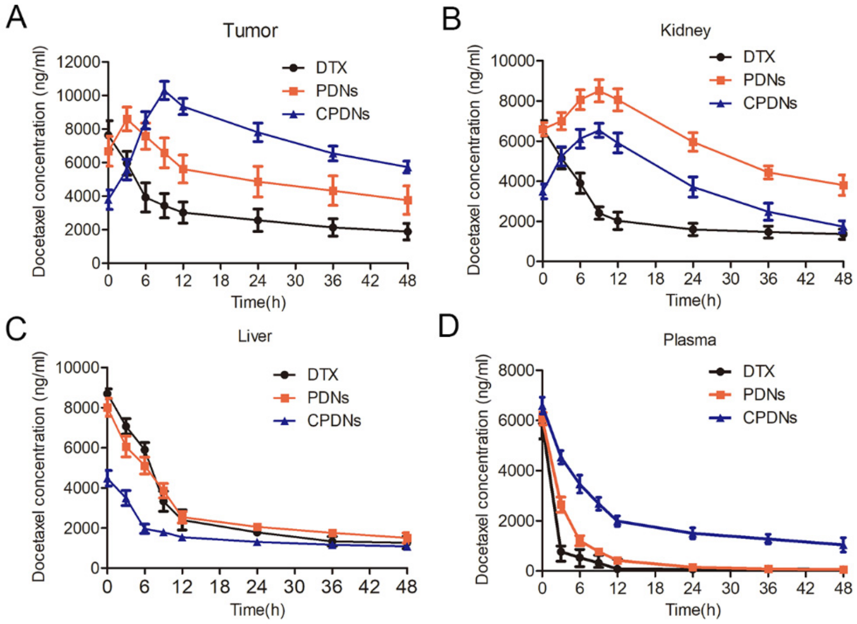

Figure S1: DTX encapsulation rate in CPDNs with various concentration loading (n = 3).

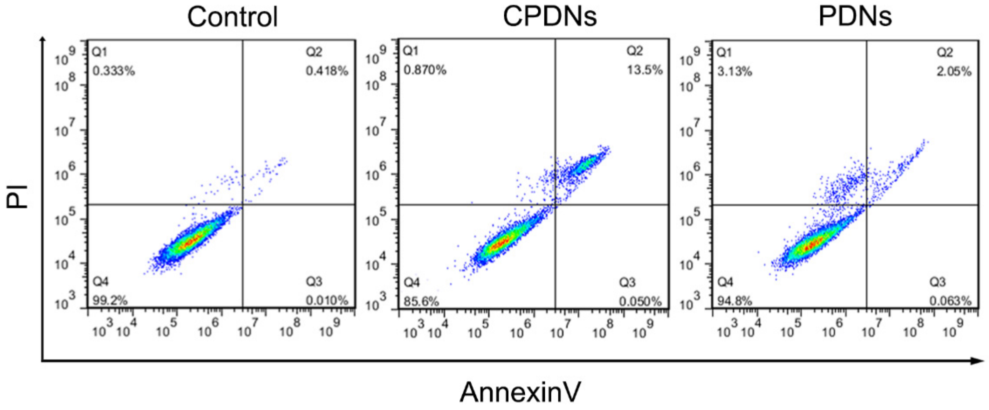

Figure S2: Flow cytometry analysis of cell apoptosis with Annexin V (V-FITC)-propidium (PI) double staining treated with different groups.

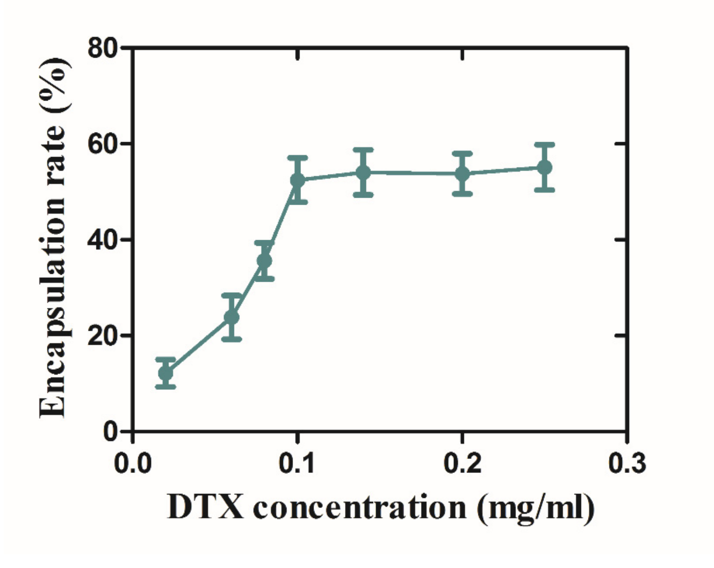

Figure S3: (A)DTX distribution in tumor following injection of DTX, PDNs and CPDNs during 48h. (B) Kidney. (C)Liver. (D)Plasma.
